# Second Primary Malignancy Risk in Multiple Myeloma from 1975 to 2018

**DOI:** 10.3390/cancers14194919

**Published:** 2022-10-07

**Authors:** Jing Wang, Chenglan Lv, Min Zhou, Jing-Yan Xu, Bing Chen, Yuan Wan

**Affiliations:** 1 Department of Oncology and Hematology, Yizheng Hospital of Nanjing Drum Tower Hospital Group, Yizheng 211400, China; 2 Department of Hematology, The Affiliated Drum Tower Hospital of Nanjing University Medical School, 321 Zhongshan Road, Nanjing 210008, China; 3 The Pq Laboratory of BiomeDx/Rx, Department of Biomedical Engineering, Binghamton University SUNY, Binghamton, NY 13902, USA

**Keywords:** myeloma, second primary malignancy, SEER, risk, survival

## Abstract

**Simple Summary:**

Multiple myeloma (MM) is the second most common hematological malignancy with median age of diagnosis between 65–70 years. Since the introduction of multiple novel agents in MM management, survival times of MM patients continue to improve. Longer survival in patients with MM may be associated with increased risks of developing second primary malignancies (SPM). In this study, we investigated the risk factors associated with SPM and survival among MM survivors from 1975 to 2018. Age, race, and sex were important factors for the risk of SPM. Moreover, after SPM development, MM patients had a statistically significant 1.4-fold increased risk of death than MM patients without SPM. Site- and time-specific surveillance strategies surveillance strategies should be recommended to monitor SPM in high-risk MM patients.

**Abstract:**

As the survival times for multiple myeloma (MM) patients continue to extend, the risk of a second primary malignancy (SPM) among MM survivors has become a topic of increasing concern within the medical community. The Surveillance, Epidemiology, and End Results (SEER) 9 Registry Database was used to evaluate the risk and survival of SPM among MM survivors from 1975 to 2018. The standardized incidence ratio (SIR), absolute excess risk (AER), and cumulative incidence (CMI) of SPM for MM risk were calculated. Survival and the CMI were estimated by using hazard ratios (HRs). Subgroup analyses were performed according to race, sex, age, time of myeloma diagnosis, and the SPM site. A total of 43,825 cases were recorded with the initial diagnosis of MM from 1975 to 2018. A total of 3101 (7.1%) patients developed 3407 SPMs. Solid tumors were decreased in patients with MM (SIR = 0.93; 95% CI = 0.90–0.97) compared to the general population, whereas the risk of hematological malignancy was increased (SIR = 1.90; 95% CI = 1.72–2.10). Taking death as a competing event, the CMI of SPM in the whole population was 7.38% at 10 years (6.11% solid and 1.27% hematologic). Factors associated with SPM occurrence were age, sex, race, and time of MM diagnosis. The survival of SPM patients from MM diagnosis was longer than that of patients without SPM (HR = 0.67, 95% CI = 0.58–0.63). The median survival time was 17 months from SPM diagnosis and 34 months from MM diagnosis (HR = 1.4, 95% CI = 1.35–1.46). Age, race, and sex were important factors for the risk of SPM. Site- and time-specific surveillance strategies should be recommended to monitor SPM in high-risk MM patients.

## 1. Introduction

Although multiple myeloma (MM) remains an incurable plasma cell neoplasia, the outcomes of patients with MM have improved dramatically in the twenty-first century due to the abundant use of autologous stem cell transplants (ASCTs) and multiple novel agents, including proteasome inhibitors (PIs) and immunomodulatory agents (IMiDs) [1], with the current median survival ranging from 5 to 8 years [2,3]. With the extension of the survival times of MM patients, the development of second primary malignancy (SPM) has become an important issue in myeloma management [4,5,6]. More than 30 years after adopting ASCT, high-dose melphalan as conditioning followed by ASCT has been administered as a standard regimen for younger MM patients [7]. A relationship between alkylating agent exposure and the risk of SPM in MM was reported in the 1970s [8,9]. The combination of melphalan and cyclophosphamide presents an effect on the pathogenesis of hematological SPM [10]. In addition, a higher risk of SPM has been noted in clinical trials that addressed the role of lenalidomide maintenance in both transplant and non-transplant populations [11,12,13]. A meta-analysis comparing the occurrence of SPM in patients with newly diagnosed MM exposed to lenalidomide found no difference in the incidence of solid SPM, but a rising risk of developing hematological SPM was observed, especially in the population exposed to lenalidomide combined with oral melphalan [14]. However, the 10-year mortality due to SPM in patients with MM undergoing ASCT was low [6], and lenalidomide maintenance was associated with a 26% reduction in mortality risk [15], suggesting that the benefits outweigh the risks due to SPM. A recent Swedish population-based study, including almost 27,000 MM patients diagnosed between 1958 and 2011, confirmed that patients with SPM had a statistically significant 2.3-fold increased risk of death versus patients without SPM [16]. Jonsdottir et al. also found that MM patients with AML/MDS had a 70% higher risk of dying than those with de novo AML/MDS [16].

Our research aims to disseminate the knowledge of SPM in newly diagnosed MM through the Surveillance, Epidemiology, and End Results (SEER) 9 Registry Database. In this study, we considered the following questions: (a) Do modern therapies increase the risk of SPM in MM? (b) What are the risk factors for SPM in MM? (c) Does SPM affect the survival of MM? It is hoped that the descriptive findings of this study will provide a greater understanding to assist physicians in the management of multiple myeloma.

## 2. Methods

### 2.1. Data Source

We utilized patient information, including patient demographic characteristics, tumor characteristics, diagnosis time, and survival date, from the SEER 9 Registry Database (1973–2018), representing approximately 9% of the United States population. The MM cases that were not the primary tumor and a 2-month latency from the first diagnosis of MM exclusion were inclusion criteria set to prevent ascertainment bias. The study adhered to the STROBE checklist for observational research [17].

### 2.2. Statistical Analysis

The standardized incidence ratio (SIR) and absolute excess risk (AER) were calculated to measure the risk of SPM by using SEER*Stat (version 8.3.9). The referent rate for the expected number of cancers was calculated for a reference cohort among the US general population matched for age, gender, race, and year of diagnosis. The 95% confidence interval (CI) for SIR was calculated assuming a Poisson distribution for the observed number of SPM. Taking death as a competing event, the Fine–Gray proportional hazards model was established to calculate the subdistribution hazard ratios (HR) to identify possible risk factors for SPM development using the R package “cmprsk”. Survival time was measured from the diagnosis date of the MM or SPM to the date of death from any cause. Survival analyses were performed by the Kaplan–Meier method and the log-rank test. A Cox, proportional hazards model was used to calculate the HR and 95% CI to compare survival according to the categories of the variables. Subgroup analyses were performed according to age strata (10-year age groups), race (white, black, other), sex, type of SPM (solid, hematologic), and latency period (2–5 months, 6–11 months, 12–59 months, 60–119 months, ≥120 months). To speculate about the impact of anti-MM therapy on SPM risk, we estimated the risk by the time of MM diagnosis under the assumption, following similar methods from previously published population-based studies [18,19]. Categories include the 1970s and 1980s, representing the use of alkylating agents; the 1990s, representing the introduction of ASCT; the 2000s, representing the wide use of ASCT and introduction of IMiDs and proteasome inhibitors; and the 2010s, representing the wide use of IMiDs and proteasome inhibitors and introduction of monoclonal antibody (anti-CD38). Statistical analyses were performed with R software, SPSS statistics (version 19) and GraphPad Prism (version 8.0). All tests were two-sided, and a *p* value < 0.05 was considered statistically significant.

## 3. Results

A total of 43,825 cases were recorded as primary MM from 1975 to 2018. A total of 3101 (7.1%) patients developed 3407 SPMs. The median age at MM diagnosis was 67.0 years old, and the median age at SPM diagnosis was 71.0 years old. Most patients (74.2%) were white. More than half of the patients (60.5%) were male. The median latency for SPM was 44 months (range 3–407 months). The median latency for SPM was similar in the earlier four eras: 45.5 months (range 3–405 months), 46 months (range 3–407 months), 50.5 months (range 3–344 months), and 48 months (range 3–213 months) for those diagnosed in the 1970s, 1980s, 1990s, and 2000s, respectively. In contrast, the median latency for SPM in patients diagnosed in the 2010s was 26 months (range 3–119 months), related to the short follow-up time. Patient characteristics are summarized in Table 1.

### 3.1. Distribution of SPM Sites

Solid tumors were more commonly diagnosed than hematologic malignancies, with prostate, lung and bronchus, breast, urinary bladder, and melanoma being the most common (Table 1). In hematologic SPM, the main types are acute myeloid leukemia (AML), non-Hodgkin’s lymphoma (NHL), myelodysplastic syndrome (MDS), chronic myeloid leukemia (CML), and acute lymphocytic leukemia (ALL). Leukemia was the most common hematologic malignancy, accounting for 7.34% of SPMs. The AML was the most common leukemia diagnosed, accounting for 63.20%. The MDS was captured starting in 2002, accounting for 23.67% of hematologic SPMs.

### 3.2. Risk of SPM

**Figure 1:**
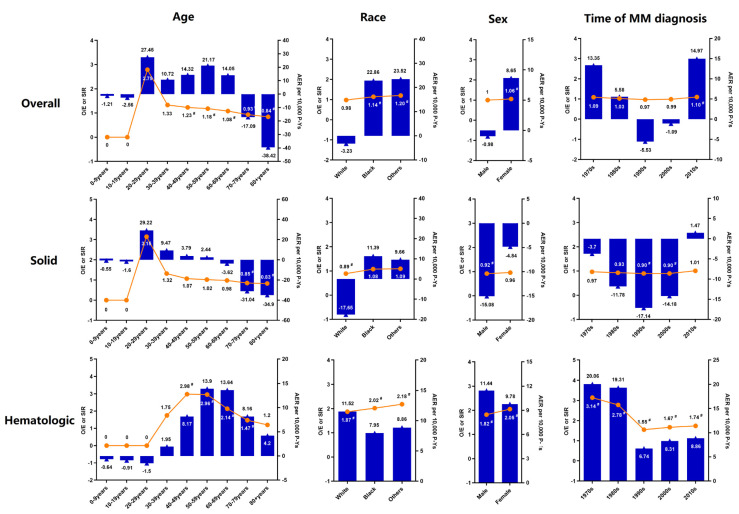
Risk of developing overall, solid, hematological SPM among patients who were diagnosed with MM as a primary cancer according to sex, race, age, and time of MM diagnosis. #, *p* < 0.05.

No significant difference was observed in the risk of overall SPM among MM patients compared to the general population (SIR = 1.02; 95% CI = 0.99–1.06), with heterogeneity by SPM type (Figure 1). However, we observed a significantly increased risk of SPM in patients diagnosed in the 2010s (SIR = 1.10; 95% CI = 1.02–1.18, AER = 14.97) (Figure 1). After 60 months of latency, a significant increased risk of SPM was observed, especially during the 2010s. Women had a significantly higher risk of SPM (SIR = 1.06; 95% CI = 1.00–1.13, AER = 8.65). A statistically significant increased risk of SPM was observed in patients aged 40 to 69 years compared with patients younger than 40 years; however, a statistically significant decreased risk of SPM was observed when patients were > = 70 years old compared with patients aged 40 to 69 years. There was a higher risk of SPM in nonwhite populations (Black: SIR = 1.14; 95% CI = 1.05–1.23, AER = 22.86, other: SIR = 1.20; 95% CI = 1.02–1.40, AER = 23.52) (Figure 1).

**Figure 2:**
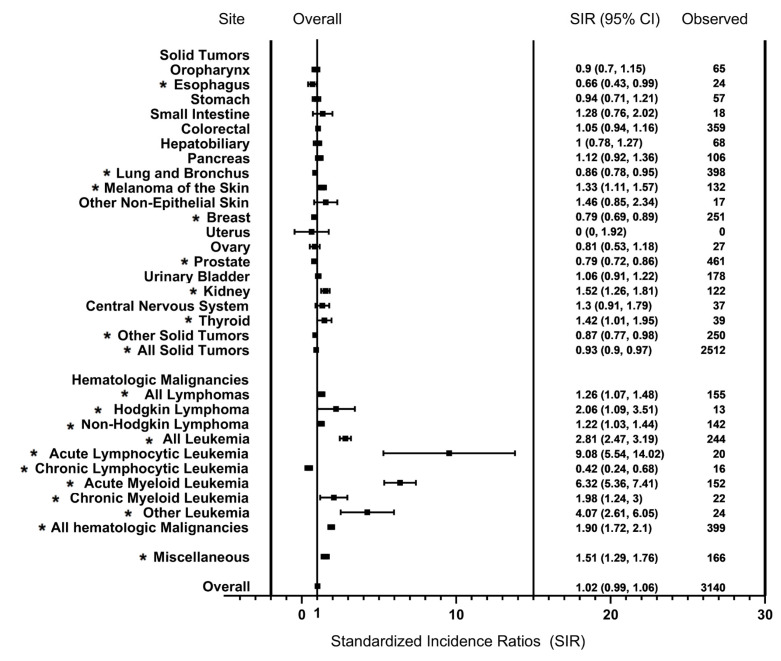
Site-specific risk of developing SPM among patients diagnosed with MM as a primary cancer. *, *p* < 0.05.

A significantly decreased risk of solid SPM was shown (SIR = 0.93; 95% CI = 0.90–0.97). There was a lower risk of esophageal (SIR = 0.66; 95% CI = 0.43–0.99), lung/bronchus (SIR = 0.86; 95% CI = 0.78–0.95), breast (SIR = 0.79; 95% CI = 0.69–0.89), and prostate (SIR = 0.79; 95% CI = 0.72–0.86) cancers, but a higher risk of melanoma (SIR = 1.33; 95% CI = 1.11–1.57), kidney/renal pelvis (SIR = 1.52; 95% CI = 1.26–1.81), and thyroid (SIR = 1.42; 95% CI = 1.01–1.95) SPM (Figure 2). However, there was no significant difference in the risk of solid SPM in patients diagnosed in the 2010s (SIR = 1.01; 95% CI = 0.93–1.10). The SIRs for solid SPM decreased with increasing age when patients were older than 70 years old. There was a significantly decreased risk of solid SPM in men (SIR = 0.92; 95% CI = 0.87–0.96). White patients had a significantly lower risk of solid SPM (SIR = 0.89; 95% CI = 0.85–0.93) (Figure 1 and Table S1).

We also showed a statistically significant increased risk of blood cancer in MM (SIR = 1.90; 95% CI = 1.72–2.10). Chronic lymphocytic leukemia (CLL) was the only hematologic cancer that MM patients were significantly less likely to develop (SIR = 0.42; 95% CI = 0.24–0.68). The SIRs were 1.26 (95% CI = 1.07–1.48) for lymphoma, 9.08 (95% CI = 5.54–14.02) for ALL, and 6.32 (95% CI = 5.36–7.41) for AML; the excess risk of hematologic SPM was primarily due to AML and ALL. The change in risk across latency periods or age strata was more heterogeneous among hematological malignancies. During the 2010s, a significantly increased risk was observed for SPM after 60 months of latency, and we also showed an overall twofold to threefold increased risk for blood cancer in patients aged between 50 and 69 years. Women had a higher risk of hematological malignancy than men (female: SIR = 2.05; 95% CI = 1.75–2.40, male: SIR = 1.82; 95% CI = 1.59–2.06). White patients had a lower risk of blood cancer than black patients (White: SIR = 1.87; 95% CI = 1.67–2.09, Black: SIR = 2.02; 95% CI = 1.52–2.64) (Figure 1). More detailed SIR results are summarized in Table 2.

### 3.3. Cumulative Incidence and Risk Factors for SPM

**Figure 3:**
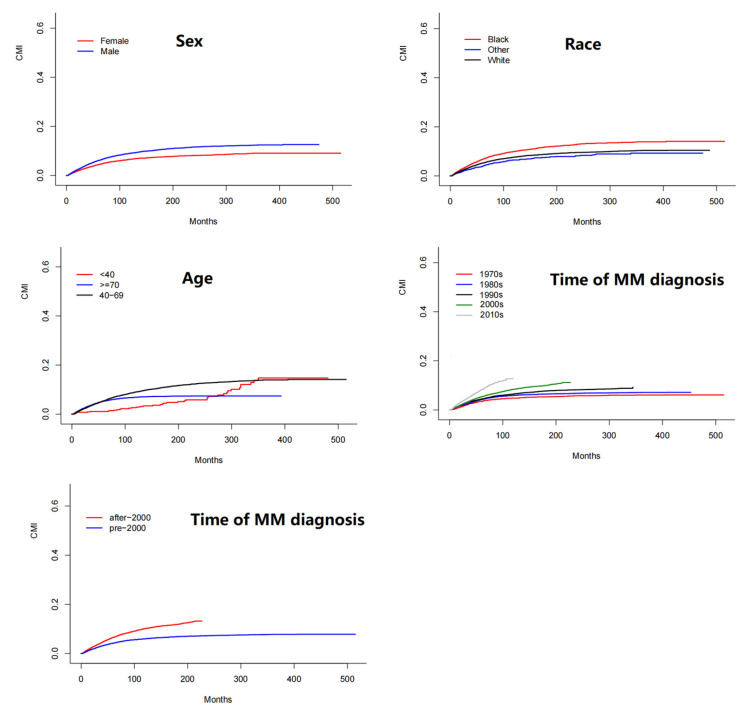
Estimates of cumulative incidence of developing SPM according to sex, race, age, and time of MM diagnosis, taking death as a competing event.

The cumulative incidence of SPM in the whole population was 7.38% at 10 years (6.11% solid and 1.27% hematologic), taking death as a competing event. Factors associated with SPM occurrence after the initial diagnosis were age, sex, race, and the year of MM diagnosis (Figure 3). Significantly higher CMI was observed in patients 40–69 years old than in younger patients (HR = 1.65, 95% CI = 1.21–2.23) (Table 3). A similar CMI was observed between patients younger than 40 years and patients older than 70 years (HR = 1.11, 95% CI = 0.82–1.51). The CMI of black patients was higher than that of white patients (HR = 1.33, 95% CI = 1.22–1.45). The CMI of female patients was significantly lower than that of male patients (HR = 0.72, 95% CI = 0.67–0.77). Patients diagnosed after 2000 had a higher CMI than patients diagnosed before 2000 (HR = 1.31, 95% CI = 1.23–1.40). There was a borderline difference in CMI between patients diagnosed in the 2010s and 2000s (HR = 1.10, 95% CI = 1.00–1.21, *p* = 0.053). Furthermore, ALL (HR = 6.15, 95% CI = 1.92–19.7), kidney/renal pelvis (HR = 2.08, 95% CI = 1.38–3.15) and thyroid (HR = 2.18, 95% CI = 1.15–4.10) SPM significantly increased in the 2010s compared with the 2000s.

### 3.4. Survival

**Figure 4:**
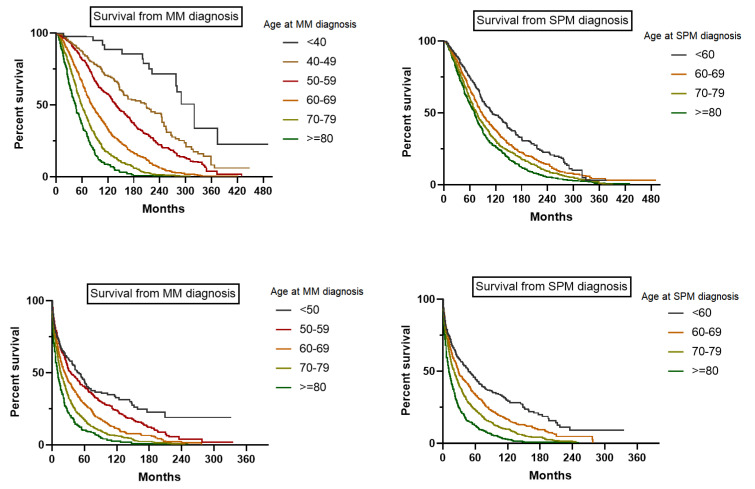
Estimates the impact of age on survival of MM patients developing SPM.

The survival of SPM patients from MM diagnosis was longer than that of patients without SPM (HR = 0.67, 95% CI = 0.58–0.63). The median survival was 17 months after SPM diagnosis and 34 months after MM diagnosis for MM patients without SPM (HR = 1.4, 95% CI = 1.35–1.46). The median survival time of patients without SPM increased from 26.0 months to 62.0 months between the 1970s and 2010s. The median survival time from SPM diagnosis increased from 12.0 months to 27.0 months between the 1970s and 2010s. Patients with SPM whose MM was diagnosed in the 2010s had a worse survival than patients diagnosed in the 2000s (HR = 1.44, 95% CI = 1.25–1.67, years from SPM diagnosis). However, patients without SPM diagnosed in the 2010s had a more pronounced survival advantage than patients diagnosed in the 2000s (HR = 0.69, 95% CI = 0.67–0.72). The survival times of SPM patients after SPM diagnosis in the 2010s and 2000s were similar (HR = 0.93, 95% CI = 0.82–1.06, years from SPM diagnosis). Age was related to the survival of patients with SPM (Figure 4 and Figure S1).

## 4. Discussion

Patients with a history of other malignancies were frequently excluded from cancer randomized clinical trials [20,21], and little is known about the long-term prognosis for those patients. Therefore, understanding the nature and characteristics of SPM and identifying high-risk patients developing SPM are of great importance. This is the largest, most detailed, and most recent population-based study to unbiasedly investigate the risk and survival of SPM in MM survivors. We noted significant heterogeneity in SPM risk by site, age, sex, race, and time of MM diagnosis.

The 10-year CMI was 6.74% for developing solid tumor SPM, and 1.43% for hematologic malignancies, similar to the CMI reported in a previous study [6]. The etiology of SPM is multifactorial, likely resulting from a combination of various intrinsic and extrinsic risk factors. Our study reported a decreased risk of solid SPM among MM patients (SIR = 0.93; 95% CI = 0.90–0.97) compared with the general population but noted an increased incidence of hematologic malignancies (SIR = 1.90; 95% CI = 1.72–2.10), similar to findings from other population studies [19,22,23,24,25,26]. A decrease or regional differences in screening rates for solid tumors after MM diagnosis could be a possible explanation for the decreased risk observed in solid tumor SPM [19]. The incidence of developing hematologic SPMs was relatively high within 12 months of the MM diagnosis and increased significantly compared to the general population. We showed a 6.32-fold increased risk of AML following MM diagnosis, similar to the risk reported in a previous study [27]. However, our study also found a significantly increased risk of ALL and HL.

Age is a known risk factor for cancer development in the general population [28]. Chakraborty et al. reported advanced age as a risk factor for SPM [24], while Razavi reported increasing age to be protective [19]. The overall risk of developing SPM significantly decreased with advanced age, and this pattern was most prominent in solid tumors (70–79 years: SIR = 0.85; 95% CI = 0.79–0.92, ≥80 years: SIR = 0.84; 95% CI = 0.74–0.94). A significantly increased risk of hematologic SPM was noted for the 50–69 age population. There was no difference in the incidence of solid and hematologic SPM between patients younger than 40 years and patients older than 70 years. Patients aged 40–69 years had a significantly higher incidence of solid and hematologic SPM than patients of other ages, which may be associated with abundant use of ASCT at this age stage and reduction in lifespan in the older age population. The elderly population may not be suitable for high-dose chemotherapy, which has been shown to increase the incidence of solid SPM in a population study [19].

Sex plays an important role in the incidence of cancers, with males generally having a lower incidence than females [29]. We found that women had a significantly higher risk of SPM than expected (female: SIR = 1.06; 95% CI = 1.00–1.13). A decreased risk of solid SPM was also observed (SIR = 0.92; 95% CI = 0.87–0.96) among male patients compared with the general population, which may be associated with a reduced risk of prostate cancer (SIR = 0.79; 95% CI = 0.72–0.86). We also found that females had a 28% lower incidence of SPM than males (HR = 0.72; 95% CI = 0.67–0.77). Similar results were observed in solid and hematologic SPM, which provides strong evidence that the fundamental biology of sex differences affects cancers of all types. Modified sex hormone levels could explain the decreased risk of some hormone-related solid SPMs, such as breast and prostate cancer. Several studies have also suggested that males are associated with increased SPM incidence [24,25].

A racial disparity has been noted in multiple studies to be a risk factor for the incidence of breast [30], colorectal [31], prostate [32], prostate [33] and cervical cancer [34]. We found that the impact of the patient’s ethnic background on the risk of developing SPM among MM patients is varied. For the overall SPM analyzed together, the O/E risk was similar in the White population compared to the general population. However, the SIR was significantly decreased for solid SPM and increased for hematological malignancies. We also found that white patients had a lower incidence of overall and solid SPM than black patients. However, there was no significant difference in the incidence of hematological malignancies. Many studies have noted significant disparities in SPM incidence in MM survivors across patients of different ethnicities [24,26]. Specific SPM disease risks also differed among different races. Compared with the general population, Hispanic whites have a high risk of bone and joint cancer, and non-Hispanic whites and black individuals have a high risk of ALL.

We noted a higher SIR of hematological malignancies among all diagnosis periods. Hematologic SPM risk started at 12 months after myeloma diagnosis and then increased with time, reaching the highest risk after 5–10 years. A study in Finland of 432 MM patients treated with prolonged melphalan-based therapy between 1979 and 1985 showed a higher risk of acute leukemia [35]. Another study of 491 newly diagnosed MM patients in Japan noted a relationship between cyclophosphamide-based therapy and SPM development [36]. However, there was no significant difference in the incidence of hematologic SPM between the 1970s and 1980s. Patients in the 1990s with a 10-year CMI of 0.9% had a similar risk of hematologic SPM compared to patients in the 1980s. However, the 10-year CMI in the 2000s significantly increased to 1.62% (aHR 1.32, 95% CI 1.05–1.66). A study involving 16,331 MM between 1991 and 2013 showed that ASCT was associated with an increased risk of hematologic SPM (aHR 1.51, 95% CI 1.01–2.27) with a 10-year CMI of 2.1% among ASCT recipients [6]. We hypothesize that ASCT combined with novel agents could increase the incidence of hematological malignancies in MM survivors due to prolonged survival times and drugs such as lenalidomide. A recent meta-analysis of seven trials, totaling 3254 newly diagnosed MM patients, found that patients who received lenalidomide had an increased risk of developing hematologic SPM [14]. A combination of oral melphalan and lenalidomide might be the main risk factor for hematologic SPM [14]. More effective therapy may be associated with a higher SPM incidence due to longer survival times. The common use of post-ASCT lenalidomide maintenance and the additional introduction of monoclonal antibodies in the 2010s did not increase the overall incidence of solid and hematologic SPM, which may be related to insufficient follow-up time. However, the incidence of certain tumors, such as ALL, kidney/renal pelvis and thyroid cancer, is still rising further.

This study provides further clinical and survival details on patients with MM with SPMs. We found that patients with SPM experienced better survival after initial MM diagnosis because SPM is associated with long survival. After SPM development, MM patients had a statistically significant 1.4-fold increased risk of death than MM patients without SPM, similar to a previously Swedish population-based study [16]. The inferior survival in MM patients with SPM is most likely multifactorial. One could argue that patients may not tolerate the treatment for SPM because of age or performance status. Our results showed that age is a strong prognostic factor in the prognosis of MM patients with SPM. Furthermore, MM patients with AML/MDS had an inferior prognosis than patients with de novo AML/MDS [16].

This study has several limitations that cannot be ignored. First, the SEER database lacks detailed treatment information, comorbidities, health behaviors, and genetic information, and we cannot estimate the impact of these factors on SPM. Geographic differences in screening rates for tumors, and differences in comorbidities in the different populations may account for some of the disparities in results. Second, there will be a substantial increase in MM survivors with SPM due to longer survival and follow-up, and the true results of SPM in the 2010s are subject to long-term follow-up. Third, patients with SPM who had been diagnosed ≥2 months after the diagnosis of MM in our study were included in determining whether the time node would be good enough to account for surveillance bias and immortal time bias. Therefore, more specific and long-term follow-up research is needed.

## 5. Conclusions

Our findings suggest that solid tumors were decreased in patients with MM, whereas the risk of hematological malignancy was increased; sex, race and age are risk factors for SPM; and MM patients with SPM had a worse prognosis, which was influenced by age. Site- and time-specific surveillance strategies should be recommended to monitor SPM in high-risk MM patients. Regardless, SPM risk remains a concern across all populations with prolonged prognosis.

## Figures and Tables

**Table 1:** Characteristics of SPM patients with MM.

	Year	1975–2018 (*n* = 3101)	1975–1979 (*n* = 186)	1980–1989 (*n* = 532)	1990–1999 (*n* = 752)	2000–2009 (*n* = 951)	2010–2018 (*n* = 680)
Characteristics							
Median age at MM diagnosis, years (IQR)		67.0 (59.0–74.0)	65.0 (56.75–72.0)	68.0 (61.0–74.0)	67.5 (58.0–74.0)	66.0 (58.0–73.0)	68.0 (61.0–75.0)
Median age at SPM diagnosis, years (IQR)		71.0 (65.0–78.0)	70.0 (62.75–77.25)	73.0 (67.0–79.0)	72.0 (65.0–78.0)	71.0 (64.0–78.0)	70.0 (64.0–77.0)
Median latency, months (IQR)		44.0 (19.0–83.0)	45.5 (23.75–89.0)	46.0 (19.25–82.75)	50.5 (22.0–101.0)	48.0 (22.0–89.0)	26.0 (11.0–47.0)
**Age at MM diagnosis (%)**							
20–29 years		2 (0.06)	1 (0.54)	0 (0)	1 (0.13)	0 (0)	0 (0)
30–39 years		32 (1.03)	3 (1.61)	7 (1.32)	10 (1.33)	6 (0.63)	6 (0.88)
40–49 years		168 (5.42)	13 (6.99)	27 (5.07)	62 (8.25)	49 (5.15)	17 (2.50)
50–59 years		593 (19.12)	45 (24.19)	79 (14.85)	142 (18.88)	211 (22.19)	116 (17.06)
60–69 years		1086 (35.02)	65 (34.95)	197 (37.03)	230 (30.59)	340 (35.75)	254 (37.35)
70–79 years		897 (28.93)	42 (22.58)	167 (31.39)	236 (31.38)	262 (27.55)	190 (27.94)
80+ years		323 (10.42)	17 (9.14)	55 (10.34)	71 (9.44)	83 (8.73)	97 (14.27)
**Age at SPM diagnosis (%)**							
0–9 years		0 (0)	0 (0)	0 (0)	0 (0)	0 (0)	0 (0)
10–19 years		0 (0)	0 (0)	0 (0)	0 (0)	0 (0)	0 (0)
20–29 years		0 (0)	0 (0)	0 (0)	0 (0)	0 (0)	0 (0)
30–39 years		5 (0.16)	0 (0)	1 (0.19)	0 (0)	1 (0.11)	3 (0.44)
40–49 years		55 (1.77)	6 (3.23)	6 (1.13)	16 (2.13)	10 (1.05)	17 (2.50)
50–59 years		319 (10.29)	24(12.90)	35 (6.58)	72 (9.58)	113 (11.88)	75 (11.03)
60–69 years		921 (29.70)	59 (31.72)	135 (25.37)	203 (26.99)	301 (31.65)	223 (32.79)
70–79 years		1177 (37.96)	59 (31.72)	242 (45.49)	303 (40.29)	348 (36.59)	225 (33.09)
80+ years		624 (20.12)	38 (20.43)	113 (21.24)	158 (21.01)	178 (18.72)	137 (20.15)
**Sex (%)**							
Male		1884 (60.75)	111 (59.68)	319 (59.96)	463 (61.57)	576 (60.57)	408 (60.00)
Female		1209 (39.25)	75 (40.32)	213 (40.04)	294 (38.43)	375 (39.43)	272 (40.00)
**Race (%)**							
White		2302 (74.23)	150 (80.65)	401 (75.38)	570 (75.80)	709 (74.55)	472 (69.41)
Black		626 (20.18)	31 (16.66)	111 (20.86)	141 (18.75)	189 (19.87)	154 (22.65)
Others		172 (5.55)	5 (2.69)	20 (3.76)	41 (5.45)	53 (5.58)	53 (7.79)
**Type of SPM (%)**							
Prostate		488 (14.32)	26 (12.44)	81 (14.04)	117 (14.10)	175 (16.51)	89 (12.18)
Lung and Bronchus		420 (12.33)	30 (14.35)	80 (13.86)	104 (12.53)	117 (11.04)	89 (12.18)
Breast		272 (7.98)	11 (5.26)	50 (8.67)	64 (7.71)	87 (8.21)	60 (8.21)
Urinary Bladder		187 (5.49)	19 (9.09)	44 (7.63)	40 (4.82)	50 (4.72)	34 (4.65)
Melanoma		160 (4.70)	5 (2.39)	12 (2.08)	33 (3.98)	65 (6.13)	45 (6.16)
Kidney and Renal Pelvis		131 (3.85)	4 (1.91)	14 (2.43)	25 (3.01)	36 (3.40)	52 (7.11)
AML		158 (4.64)	19 (9.09)	39 (6.76)	24 (2.89)	45 (4.25)	31 (4.24)
ALL		22 (0.65)	3 (1.44)	0 (0)	6 (0.72)	3 (0.28)	10 (1.37)
CML		23(0.74)	2(1.08)	2(0.04)	10(1.33)	5(0.53)	4(0.59)
MDS		102 (2.99)	0 (0)	0 (0)	26 (3.13)	48 (4.53)	28 (3.83)
NHL—Nodal		107 (3.14)	8 (3.83)	20 (3.47)	25 (3.01)	36 (3.40)	18 (2.46)
NHL—Extranodal		69 (2.03)	1 (0.48)	9 (1.56)	14 (1.69)	28 (2.64)	17 (2.33)

IQR, interquartile range.

**Table 2:** Standardized incidence ratios (SIR) of hematological second primary malignancies (SPMs).

**Characteristics**	**1975–2018** **SIR (95% CI)**	**1970s** **SIR (95% CI)**	**1980s** **SIR (95% CI)**	**1990s** **SIR (95% CI)**	**2000s** **SIR (95% CI)**	**2010s** **SIR (95% CI)**
	1.90 (1.72–2.10) ^#^	3.14 (2.18–4.36) ^#^	2.78 (2.23–3.43) ^#^	1.55 (1.22–1.95) ^#^	1.67 (1.39–2.00) ^#^	1.74 (1.39–2.16) ^#^
**Latency period** (Months)						
2–5	1.78 (1.19–2.55) ^#^	0.00 (0.00–4.32)	2.83 (1.14–5.84) ^#^	2.06 (0.83–4.25)	2.48 (1.24–4.44) ^#^	0.77 (0.21–1.98)
6–11	0.97 (0.60–1.48)	0.88 (0.02–4.93)	1.83 (0.67–3.99)	0.22 (0.01–1.23)	0.50 (0.10–1.46)	1.47 (0.70–2.70)
12–59	1.63 (1.39–1.89) ^#^	2.54 (1.35–4.34) ^#^	2.19 (1.51–3.07) ^#^	1.06 (0.67–1.59)	1.59 (1.19–2.09) ^#^	1.64 (1.22–2.17) ^#^
60–119	2.87 (2.40–3.42) ^#^	7.26 (4.15–11.80) ^#^	4.38 (2.86–6.42) ^#^	2.59 (1.69–3.79) #	1.95 (1.40–2.66) ^#^	3.39 (2.07–5.23) ^#^
≥120	2.25 (1.68–2.96)	2.70 (0.88–6.30)	3.30 (1.85–5.44)	2.08 (1.23–3.28)	1.71 (0.91–2.93)	NA
**Age** (Years)						
0–9	0.00 (0.00–16402.87)	0.00 (0.00–0.00)	0.00 (0.00–16402.87)	0.00 (0.00–0.00)	0.00 (0.00–0.00)	0.00 (0.00–0.00)
10–19	0.00 (0.00–11395.49)	0.00 (0.00–0.00)	0.00 (0.00–0.00)	0.00 (0.00–0.00)	0.00 (0.00–47317.55)	0.00 (0.00–15010.46)
20–29	0.00 (0.00–52.58)	0.00 (0.00–270.34)	0.00 (0.00–232.66)	0.00(0.00–200.09)	0.00 (0.00–236.48)	0.00 (0.00–556.75)
30–39	1.75 (0.21–6.32)	10.28 (0.26–57.27)	3.45 (0.09–19.22)	0.00 (0.00–11.05)	0.00 (0.00–11.32)	0.00 (0.00–37.93)
40–49	2.98 (1.89–4.46) ^#^	5.70 (1.18–16.65) ^#^	3.90 (1.27–9.10) ^#^	3.39 (1.46–6.68) ^#^	1.96 (0.64–4.57)	1.98 (0.24–7.17)
50–59	2.96 (2.38–3.64) ^#^	4.58 (2.10–8.70) ^#^	3.10 (1.69–5.19) ^#^	2.19 (1.25–3.56) ^#^	2.98 (2.06–4.16) ^#^	3.24 (1.89–5.19) ^#^
60–69	2.14 (1.80–2.53) ^#^	3.20 (1.60–5.73) ^#^	3.52 (2.45–4.90) ^#^	1.32 (0.79–2.06)	1.94 (1.39–2.62) ^#^	2.07 (1.39–2.95) ^#^
70–79	1.47 (1.20–1.77) ^#^	2.74 (1.31–5.03) ^#^	2.29 (1.49–3.39) ^#^	1.22 (0.76–1.87)	1.35 (0.92–1.90)	1.09 (0.65–1.70)
80+	1.20 (0.86–1.64)	0.68 (0.02–3.79)	1.60 (0.64–3.30)	1.65 (0.82–2.95)	0.53 (0.19–1.15)	1.60 (0.89–2.64)
**Race**						
White	1.87 (1.67–2.09) ^#^	3.31 (2.27–4.68) ^#^	2.65 (2.06–3.35) ^#^	1.35 (1.02–1.76) ^#^	1.73 (1.41–2.09) ^#^	1.75 (1.35–2.22) ^#^
Black	2.02 (1.52–2.64) ^#^	2.36 (0.49–6.90)	3.5 (1.91–5.87) ^#^	2.22 (1.18–3.80) ^#^	1.24 (0.62–2.22)	1.95 (1.04–3.34) ^#^
Other	2.18 (1.31–3.41) ^#^	0.00 (0.00–16.05)	3.41 (0.70–9.96)	3.99 (1.60–8.21) ^#^	1.94 (0.71–4.21)	1.09 (0.23–3.20)
**Sex**						
Female	2.05 (1.75–2.40) ^#^	2.98 (1.63–5.00) ^#^	2.82 (1.97–3.90) ^#^	1.87 (1.30–2.62) ^#^	1.74 (1.27–2.33) ^#^	1.88 (1.28–2.67) ^#^
Male	1.82 (1.59–2.06) ^#^	3.25 (2.01–4.96) ^#^	2.75 (2.05–3.62) ^#^	1.36 (0.98–1.84) ^#^	1.63 (1.28–2.05) ^#^	1.67 (1.24–2.18) ^#^

# Statistically significant.

**Table 3:** Significant risk factors associated with the development of SPM, estimated by the Fine–Gray subdistribution hazards model.

Factors	HR and 95% CI	*p*
**Age at MM diagnosis**		
≥70 vs. <40–69	0.67 (0.63–0.72)	4.3 × 10^−10^
≥70 vs. <39	1.11 (0.82–1.51)	0.510
40–69 vs. <39	1.65 (1.21–2.23)	0.0014
**Sex**		
Female vs. Male	0.72 (0.67–0.77)	0
**Race**		
Black vs. White	1.33 (1.22–1.45)	3.9 × 10^−11^
Other vs. White	0.80 (0.69–0.93)	3.3 × 10^−3^
Other vs. Black	0.60 (0.51–0.71)	1.1 × 10^−9^
**Time of MM diagnosis**		
post-2000 vs. pre-2000	1.31 (1.23–1.40)	1.8 × 10^−15^
2010s vs. 2000s	1.10 (1.00–1.21)	5.3 × 10^−2^
2010s vs. 1990s	1.24 (1.13–1.37)	1.6 × 10^−5^
2010s vs. 1980s	1.47 (1.32–1.64)	3.6 × 10^−12^
2010s vs. 1970s	1.73 (1.48–2.01)	2.7 × 10^−12^
2000s vs. 1990s	1.13 (1.04–1.24)	6.5 × 10^−3^
2000s vs. 1980s	1.34 (1.21–1.48)	1.2 × 10^−8^
2000s vs. 1970s	1.57 (1.36–1.82)	1.7 × 10^−9^
1990s vs. 1980s	1.18 (1.07–1.32)	1.8 × 10^−3^
1990s vs. 1970s	1.39 (1.19–1.61)	2.0 × 10^−5^
1980s vs. 1970s	1.17 (1.00–1.37)	4.7 × 10^−2^

## Data Availability

All data generated during this study are included in this article. The datasets supporting the conclusions of this article are available in SEER database: https://seer.cancer.gov/, accessed on 10 October 2021.

## Supplementary Materials

The following supporting information can be downloaded at https://www.mdpi.com/article/10.3390/cancers14194919/s1. Table S1. Standardized incidence ratios (SIR) of solid second primary malignancies (SPM). Figure S1. KM curve for OS. (A) Survival time from MM diagnosis; (B) Survival time from SPM diagnosis.

## Abbreviations

AER, absolute excess risk; ALL, acute lymphocytic leukemia; AML, acute myeloid leukemia; ASCTs, autologous stem cell transplants; CMI, cumulative incidence; CML, chronic myeloid leukemia; HRs, hazard ratios; IMiDs, immunomodulatory agents; MDS, myelodysplastic syndrome; MM, multiple myeloma; NHL, non-Hodgkin’s lymphoma; OS, overall survival; SPM, PIs, proteasome inhibitors; second primary malignancy; SEER, Surveillance, Epidemiology, and End Results; SIR, standardized incidence ratio.
