# Supplementary material for: Second Primary Malignancy Risk in Multiple Myeloma from 1975 to 2018
Source: Cancers (Basel). 2022 Oct 7;14(19):4919. doi: 10.3390/cancers14194919 (PMC9562683; doi:10.3390/cancers14194919)
Supplement: Supplementary file 1 [file cancers-14-04919-s001.zip › cancers-1891646-supplementary.pdf]

Table S1. Standardized incidence ratios (SIR) of solid second primary malignancies (SPM).

**All**	0.93 (0.90-0.97) #	0.97 (0.83-1.14)	0.93 (0.84-1.02)	0.90 (0.83-0.97) #	0.90 (0.84-0.97) #	1.01 (0.93-1.10)
**Latency period (**months**)**						
2-5	0.96 (0.83-1.10)	0.85 (0.43-1.53)	0.62 (0.40-0.94) #	1.00 (0.74-1.32)	1.09 (0.84-1.41)	1.03 (0.79-1.33)
6-11	0.94 (0.83-1.06)	0.65 (0.32-1.16)	1.14 (0.86-1.48)	0.82 (0.61-1.06)	0.73 (0.55-0.96) #	1.17 (0.94-1.43)
12-59	0.89 (0.84-0.94) #	1.01 (0.80-1.26)	0.86 (0.74-0.99) #	0.82 (0.73-0.93) #	0.88 (0.79-0.97) #	0.95 (0.85-1.06)
60-119	0.95 (0.87-1.03)	0.91 (0.61-1.30)	0.98 (0.78-1.20)	0.90 (0.75-1.08)	0.92 (0.81-1.06)	1.10 (0.86-1.38)
≥120	1.09 (0.97-1.22)	1.20 (0.83-1.68)	1.12 (0.88-1.42)	1.11 (0.92-1.34)	1.00 (0.80-1.23)	NA
**Age (**years**)**						
0-9	0.00 (0.00-19068.36)	0.00 (0.00-0.00)	0.00 (0.00-19068.36)	0.00 (0.00-0.00)	0.00 (0.00-0.00)	0.00 (0.00-0.00)
10-19	0.00 (0.00-6472.56)	0.00 (0.00-0.00)	0.00 (0.00-0.00)	0.00 (0.00-0.00)	0.00 (0.00-22960.34)	0.00 (0.00-9013.48)
20-29	3.15 (0.35-11.38)	9.97 (1.12-36.01) #	0.00 (0.00-18.53)	0.00 (0.00-36.85)	0.00 (0.00-36.94)	0.00 (0.00-98.85)
30-39	1.32 (0.77-2.12)	0.85 (0.01-4.72)	1.15 (0.31-2.94)	1.24 (0.40-2.90)	0.93 (0.19-2.70)	4.40 (1.18-11.27) #
40-49	1.07 (0.88-1.29)	1.28 (0.61-2.36)	0.97 (0.57-1.55)	1.25 (0.89-1.70)	0.99 (0.69-1.38)	0.81 (0.39-1.49)
50-59	1.02 (0.93-1.12)	1.20 (0.85-1.63)	0.91 (0.71-1.16)	0.95 (0.78-1.16)	1.01 (0.86-1.18)	1.19 (0.95-1.46)
60-69	0.98 (0.92-1.05)	1.09 (0.83-1.40)	0.98 (0.83-1.14)	0.88 (0.75-1.01)	0.99 (0.88-1.12)	1.04 (0.90-1.20)
70-79	0.85 (0.79-0.92) #	0.60 (0.41-0.85) #	0.94 (0.80-1.10)	0.86 (0.74-0.99) #	0.81 (0.71-0.93) #	0.90 (0.77-1.05)
80+	0.83 (0.74-0.94) #	1.07 (0.65-1.65)	0.74 (0.53-1.01)	0.84 (0.64-1.07)	0.70 (0.55-0.88) #	1.01 (0.81-1.25)
**Race**						
White	0.89 (0.85-0.93) #	0.95 (0.80-1.13)	0.89 (0.80-0.99) #	0.87 (0.79-0.96) #	0.85 (0.78-0.92) #	0.94 (0.85-1.04)
Black	1.08 (0.99-1.17)	0.99 (0.66-1.43)	1.06 (0.86-1.29)	1.00 (0.83-1.19)	1.13 (0.97-1.31)	1.12 (0.93-1.33)
Other	1.09 (0.91-1.30)	1.52 (0.56-3.31)	0.99 (0.54-1.66)	0.93 (0.59-1.38)	0.90 (0.64-1.24)	1.44 (1.07-1.90) #
**Sex**						
Female	0.96 (0.90-1.02)	0.97 (0.75-1.24)	0.93 (0.79-1.08)	0.94 (0.82-1.07)	0.90 (0.80-1.01)	1.09 (0.96-1.25)
Male	0.92 (0.87-0.96) #	0.98 (0.79-1.19)	0.93 (0.82-1.04)	0.87 (0.79-0.97) #	0.91 (0.83-0.99) #	0.96 (0.86-1.07)

# p<0.05.

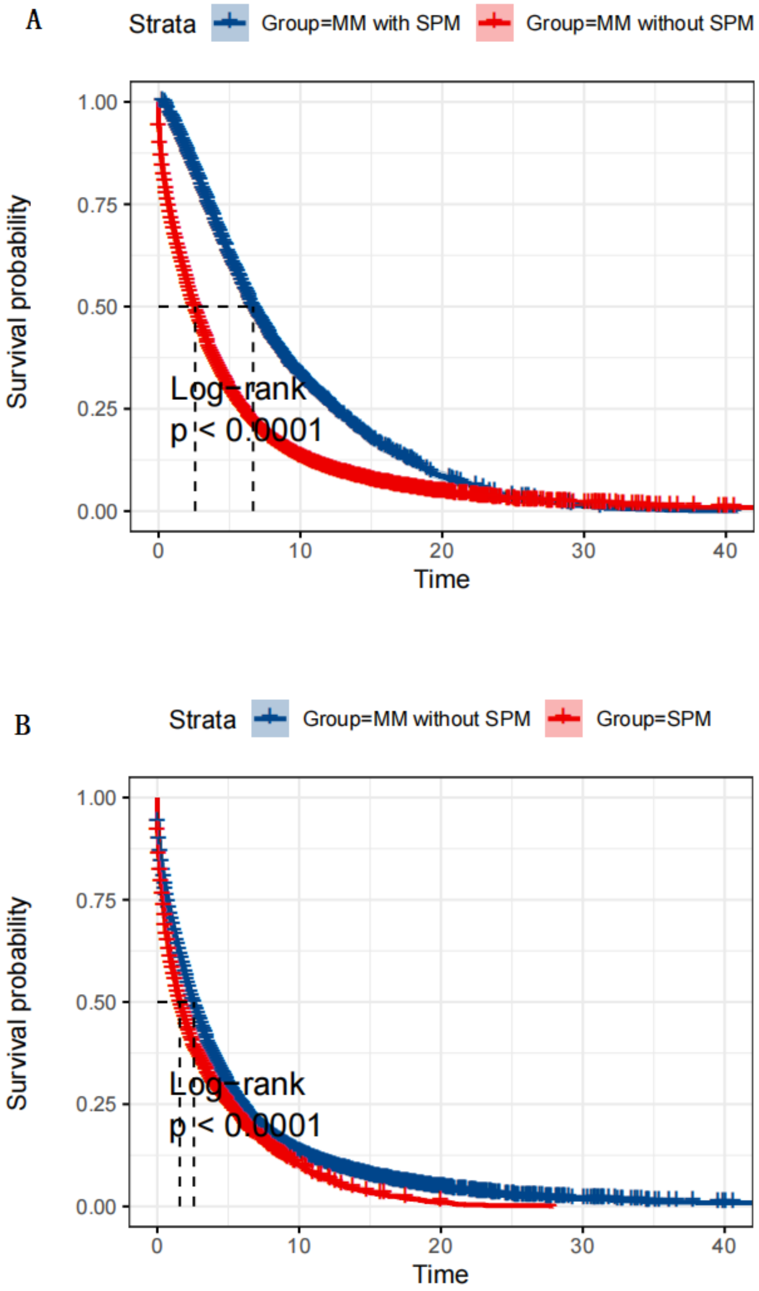

Figure S1. KM curve for OS. (**A**) Survival time from MM diagnosis; (**B**) Survival time from SPM diagnosis.
